# A hormone receptor pathway cell-autonomously delays neuron morphological aging by suppressing endocytosis

**DOI:** 10.1371/journal.pbio.3000452

**Published:** 2019-10-07

**Authors:** Claire E. Richardson, Callista Yee, Kang Shen

**Affiliations:** 1 Department of Biology, Stanford University, Stanford, California, United States of America; 2 Howard Hughes Medical Institute, Department of Biology, Stanford University, Stanford, California, United States of America; Fundacion Instituto Leloir, ARGENTINA

## Abstract

Neurons have a lifespan that parallels that of the organism and are largely irreplaceable. Their unusually long lifespan predisposes neurons to neurodegenerative disease. We sought to identify physiological mechanisms that delay neuron aging in *Caenorhabditis elegans* by asking how neuron morphological aging is arrested in the long-lived, alternate organismal state, the dauer diapause. We find that a hormone signaling pathway, the abnormal DAuer Formation (DAF) 12 nuclear hormone receptor (NHR) pathway, functions cell-intrinsically in the dauer diapause to arrest neuron morphological aging, and that same pathway can be cell-autonomously manipulated during normal organismal aging to delay neuron morphological aging. This delayed aging is mediated by suppressing constitutive endocytosis, which alters the subcellular localization of the actin regulator T cell lymphoma Invasion And Metastasis 1 (TIAM-1), thereby decreasing age-dependent neurite growth. Intriguingly, we show that suppressed endocytosis appears to be a general feature of cells in diapause, suggestive that this may be a mechanism to halt the growth and other age-related programs supported by most endosome recycling.

## Abstract 2

Neurons have a lifespan that parallels that of the organism and are largely irreplaceable. Analysis of the nematode “dauer” diapause reveals that state-dependent hormone signaling halts constitutive endocytosis, altering the localization of an actin regulator, thereby suspending dendrite morphogenesis and aging.

## Introduction

Neurons are largely irreplaceable and must therefore survive and function for the lifespan of the organism. In mammals, aging is associated with cognitive decline, structural alterations in neurons such as sprouting, and synaptic deterioration [1]. Furthermore, although diverse neuron pathologies are thought to underlie various neurodegenerative diseases, increased age is a common risk factor [2]. Whether this is due to age of the organism, the neurons, or both is not clear; still, the idea that delaying aging could postpone the onset of these diseases, as well as age-associated cognitive decline, is a major motivation for aging research.

The endomembrane system is the central mechanism by which metazoan cells interact with their organismal environment. The endosomal pathways of this system promote constant sampling of the environment—evocatively, mouse embryonic fibroblasts recycle their entire cell surface in under 15 minutes [3]. Endosomal pathways modulate activity of plasma membrane proteins, such as signaling receptors including G-Protein Coupled Receptors (GPCRs) and growth-factor receptors, adhesive molecules including integrins and cadherins, and nutrient transporters and sensors. The internalization and recycling of these proteins from the plasma membrane provides spatial and temporal mechanisms to tune fundamental processes such as cell migration, morphogenesis, survival, metabolism, and growth [4]. Furthermore, endosomes serve as signaling platforms that can recruit cytosolic proteins, and different populations of endosomes enhance or attenuate signaling [5]. For instance, Rab5-generated endosomes at the leading edge of migrating cells recruit Rac and its activator, the Rac guanine nucleotide exchange factor (GEF) T cell lymphoma Invasion And Metastasis 1 (Tiam1), thereby promoting actin dynamics to drive cell migration [6].

In neurons, endosomes likewise regulate plasma membrane proteins to modulate cellular responses to physiological changes [7], and they also perform neuron-specific functions. In neurite morphogenesis and remodeling, guidance and adhesive receptors respond to environmental cues and regulate the activity of cytoskeletal regulators, including Rac guanosine triphosphate (GTPase), which promote polymerization or de-polymerization of actin. Endosome generation and trafficking adds an additional layer of regulation to this process. Asymmetric regulation of endocytosis can control growth cone turning, and the balance of endosome generation, recycling, trafficking, and degradation via Rab GTPases impacts both the size and location of neurites [8–11]. Additionally, endocytosis is central for the specialized neuronal process of synaptic vesicle (SV) recycling, in which neurotransmitters and transmembrane synaptic proteins must be retrieved after their release in order to sustain many round of neurotransmission [12]. Outside of its role in neurotransmission, the functions of endosome generation in mature and aging neurons are not well understood.

On top of these cell-intrinsic and local regulatory mechanisms, the morphology and function of the nervous system is dramatically impacted by endocrine signaling. Sex-specific hormones organize distinctive male or female connectivity during development, govern animal behavior, and alter long-term and acute neuronal activity at the cellular level [13]. Endocrine signals that respond to experience—including stress-induced corticosteroids and nutrient-status monitors such as insulin—affect neural activity in the short term and morphology in the long term [14–18]. The importance of understanding the impact of endocrine signaling on the nervous system is underscored by the strong influence of sex, stress, and organismal metabolic signaling on neurological disorders, including depression, autism, and Alzheimer disease [19–22].

During development, *C*. *elegans* uses endocrine signaling to choose between 2 states: in favorable environmental conditions, animals undergo “reproductive development” (RD), in which they grow continuously to adulthood through 4 larval stages (L1–L4) and have a lifespan of about 2 weeks; in response to adverse environmental conditions, animals instead enter the dauer diapause—an ageless, stress-resistant alternative to the L3 larval stage—in which they can survive for months [23]. When dauer larvae sense improved conditions, they reenter RD at the L4 larval stage and exhibit a wild-type adult lifespan [23,24]. The organismal RD versus dauer decision involves the perception of signals from the environment by sensory neurons, which secrete hormones that are sensed by and subsequently induce further hormone secretion from the hypodermis and intestine, ultimately resulting in metabolic, morphologic, and functional changes throughout the organism [25,26]. Notably, these endocrine signaling pathways used for the RD-versus-dauer decision—which include insulin-like signaling, forkhead box O (FOXO)/DAF-16, and nuclear hormone receptor (NHR) DAF-12—are likewise central regulators of aging in RD. *C*. *elegans* neurons exhibit age-associated changes in morphology and cell biology in RD, which contribute to behavioral decline over the 2-week RD lifespan, yet dauer larvae are able to behave for months [27,28].

We hypothesized that neuron aging is stalled in dauer and that studying the mechanisms underlying this may provide insights into how to delay neuronal aging. To initiate this study, we used the morphology of the PVD sensory neuron as a proxy for neuron age. In worms that go through RD, the PVD neuron extends an elaborate dendritic arbor that grows continuously starting from the L2 stage [29], whereas in dauer, we find that PVD dendrite growth is reversibly arrested. We show this arrest is mediated cell-autonomously by the DAF-12 NHR pathway in dauer and that cell-specific manipulation of this pathway in RD is sufficient to delay not only PVD dendrite growth but also age-associated ectopic neurite extension in other neurons. This manipulation, which we term “dauerization,” causes a dramatic reduction in constitutive endosome generation not only in the PVD dendrite but also in cells of other tissue types, implying that suppressed endocytosis is a general feature of cells in this stalled-aging state. Intriguingly, SV endocytosis is preserved, which would permit the continued behavior of animals in dauer. Finally, we show that PVD dendritic endosomes localize TIAM-1 and present evidence supporting a model in which dendritic endosomes regulate the balance between neurite outgrowth, which is supported in RD, versus stabilization, which is favored in dauer.

## Results

### Neuron morphological aging can be cell-autonomously stalled by the dauer- and aging-related DAF-12 NHR

**Fig 1:**
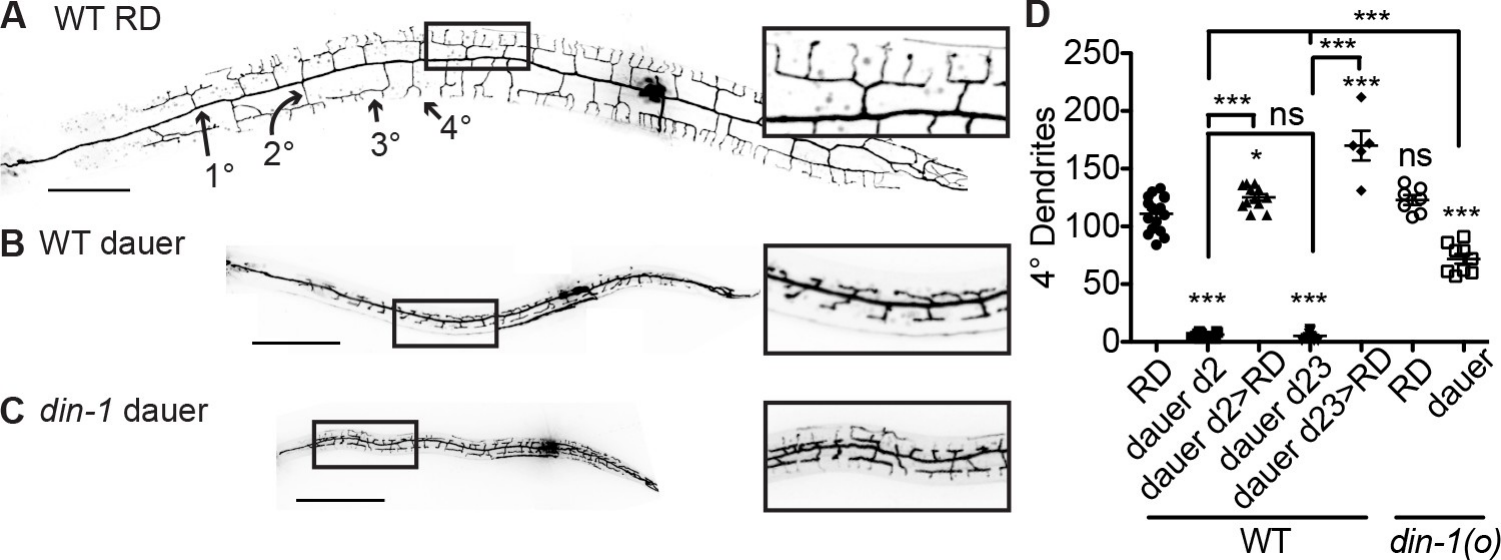
PVD dendrite exhibits a state-dependent growth arrest. (A–B) PVD dendrite morphology of wild-type animals in RD (panel A) and dauer diapause (panel B). (C) PVD dendrite fails to arrest dendrite growth in the *din-1(o)* mutant in dauer. (D) Quantification of quaternary (4°) dendrite growth in RD versus dauer in wild-type and the *din-1* mutant. ***P < 0.001, **P < 0.01, one-way ANOVA with Tukey post test. See also S1 Data. Morphology throughout this study was visualized with PVD > myr-GFP, scale = 50 μm. As the *din-1* mutant is resistant to entering dauer, a dauer-promoting mutation in another gene, *daf-2*, was added to the *din-1* genetic background; this *daf-2* allele does not affect PVD dauer dendrite arrest (S1 Fig). The *daf-2* allele is additionally present in every genetic background in which dauer animals were studied in future figures unless otherwise noted (see Materials and methods). Light artifacts due to scatter from the spinning disk were removed from images of PVD morphology here and in future figures. GFP, green fluorescent protein; myr, myristoylation; ns, not significant; RD, reproductive development; WT, wild type.

In worms that go through RD, the PVD neuron extends an elaborate dendritic arbor of orthogonally branching dendrites (Fig 1A) [29]. We observe that the PVD dendrite exhibits a reversible growth arrest in dauer at the developmental stage prior to quaternary (4°) branch outgrowth (Fig 1B and 1D). Dauer larvae develop a full set of tertiary (3°) branches but very few quaternary (4°) branches, similar to PVD morphology in an L3-stage larvae (Fig 1B) [29]. This developmental arrest persists for at least 23 days as long as the dauer state remains (Fig 1D). After dauer animals reenter RD, their PVD neurons grow 4° dendrites in higher numbers than in animals grown just in RD (Fig 1D).

We reasoned that specific signaling components involved in the organismal dauer state decision may dictate whether the PVD dendrite grows to the RD-like (with 4°s) versus dauer-like (without 4°s) morphology. The transcriptional corepressor daf-12 interacting protein (DIN-1)/split ends (SPEN) regulates organismal dauer entry but has not previously linked to neurodevelopment in *C*. *elegans* [30]. We found that DIN-1 was necessary for the dauer-dependent dendrite growth arrest (Fig 1C and 1D, S1 Fig). Consistent with the idea that *din-1* functions to instruct PVD to adopt the dauer morphology rather than to limit dendrite growth per se, the *din-1(o)* mutant exhibits a wild-type PVD dendrite morphology in RD (Fig 1D). Genes in the 2 other major endocrine signaling pathways that promote the organismal dauer decision—*daf-7/TGFβ-daf-3/SMAD* and *daf-2/insulin receptor-daf-16/FOXO*—are not required for the dauer-dependent dendrite growth arrest, indicating that the role of mediating this arrest is specific to the *din-1* pathway.

**Fig 2:**
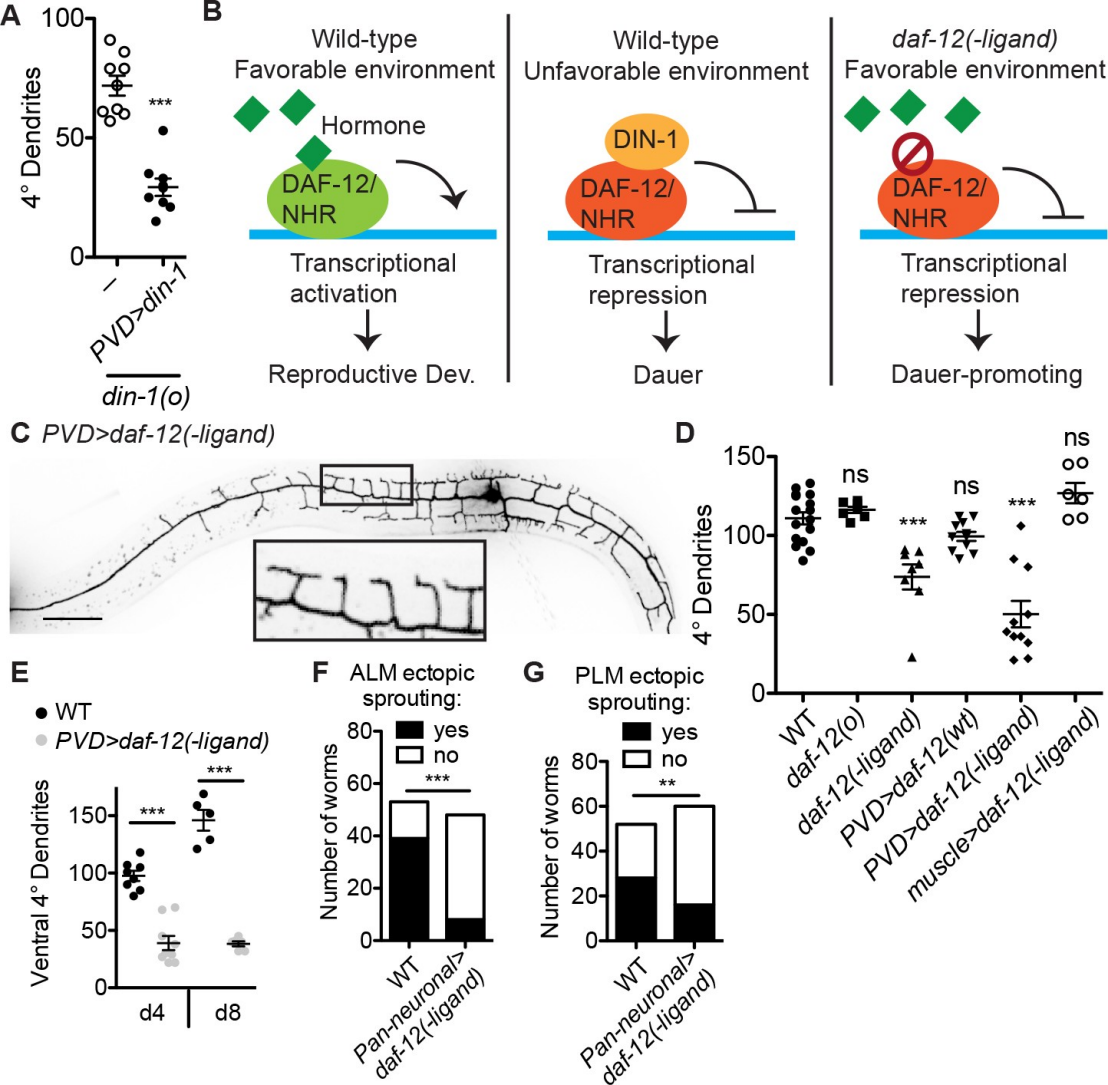
Cell-autonomous “dauerization” delays neuron morphological aging. (A) Expressing wild-type *din-1* in the PVD neuron is sufficient to rescue dauer dendrite growth arrest in the *din-1* mutant. ***P < 0.001, two-tailed *t* test. (B) DAF-12/NHR promotes RD when its hormone ligand is present and is essential for dauer entry when ligand is absent. The *daf-12(−ligand)* allele promotes dauer entry even in the presence of hormone. (C, D) PVD-specific expression of *daf-12(−ligand)* is sufficient to induce a dauer-like morphology, or “dauerization,” of PVD dendrite in animals that are otherwise in RD. *daf-12* is not required for RD, and the *daf-12(o)* appears grossly wild type. Although the *daf-12(*−*ligand)* mutant promotes dauer entry, it can be grown through RD at low temperature. ***P < 0.001, one-way ANOVA with Tukey post test. (E) PVD-specific expression of *daf-12(−ligand)* is sufficient to delay dendrite growth in aging (day 4 and day 8) animals. ***P < 0.001, two-tailed *t* test. (F) Neuronal expression of *daf-12(−ligand)* inhibits the ectopic neurite growth, characteristic of aging worms, of the ALM day 10 and PLM day 15 neurons. ***P < 0.001, chi-squared test. See also S1 Data. Scale = 50 μm. ALM, anterior lateral microtubule; DAF-12, dauer formation abnormal; DIN-1, daf-12 interacting protein; NHR, nuclear hormone receptor; ns, not significant; PLM, posterior lateral microtubule; RD, reproductive development; WT, wild type.

The cell- and tissue-specific changes downstream of the organismal signaling that induces the dauer arrest are generally not well understood [31,32]. We asked whether the PVD dauer dendrite growth arrest is mediated by DIN-1 cell-autonomously. Indeed, expressing wild-type *din-1* specifically in the PVD neuron rescues the *din-1* mutant dauer dendrite growth arrest (Fig 2A), indicating that a cell-autonomous dauer decision is necessary for the arrest.

To determine whether a cell-autonomous dauer decision could also be sufficient to induce dendrite growth arrest, we turned to the DAF-12 NHR, which binds DIN-1 to promote dauer entry [30,33,34]. As a type II nuclear receptor, DAF-12 is thought to bind DNA constitutively, repressing transcription in the absence of its hormone ligand in the dauer-promoting, adverse-environment state and activating transcription when ligand is bound in the RD-promoting, favorable-environment state [35,36](Fig 2B). Whereas *daf-12(o)* null mutants cannot enter dauer and exhibit a shortened adult lifespan in RD, the previously identified gain-of-function allele *daf-12(r273)* promotes constitutive dauer entry and extends adult lifespan in RD [34,36–38] (Fig 2B). This allele contains a missense mutation in the ligand-binding domain of DAF-12, and it is thought to lock DAF-12 in the transcriptionally repressive “−ligand” conformation [36] (Fig 2B). This type of mutation has been found in the human Thyroid Receptor beta, a homolog and functional analog of DAF-12, in patients with resistance to thyroid hormone [39]. Interestingly, mice carrying a −ligand allele of Thyroid Receptor beta show severe defects in neuronal growth and function, whereas null animals are grossly wild type, supporting the model that this hormone receptor can strongly modulate—but is not a central component of—neuronal development [40].

Whereas *daf-12(o)* mutants exhibit a PVD dendrite morphology in RD indistinguishable from that of wild type, the *daf-12(−ligand)* mutant grown in RD shows a reduction in 4° branches reminiscent of the dauer dendrite morphology (Fig 2D). Importantly, PVD-specific expression of *daf-12(−ligand)* cDNA in a wild-type genetic background is sufficient to induce a dauer-like dendrite morphology (Fig 2C and 2D). In contrast, expression of *daf-12(−ligand)* in muscles does not alter PVD dendrites, nor does overexpression of wild-type *daf-12* in PVD (Fig 2D). These genetic manipulations indicate that the dauer dendrite morphology, and perhaps by extension the dauer arrest itself, can be induced in a single neuron when the organism is otherwise in RD. This cell-autonomous genetic manipulation is hereafter referred to as “dauerization.” PVD-specific expression of a chimeric transcription factor consisting of the DAF-12 DNA binding domain attached to a trans-repressive domain, but not a trans-activating domain, can likewise inhibit dendrite morphogenesis in RD animals (S2A–S2C Fig). Taken together, these data indicate that this aging-related hormone pathway controls dendrite morphology in a cell-autonomous manner.

Because animals in the dauer state age many times slower than those in RD, we asked whether dauerization could be a method to cell-autonomously delay neuronal aging [24]. To monitor one aspect of neuronal aging, we examined the dendritic morphology of PVD. PVD form stereotyped “menorah” morphology in L3 and L4 stages. As the animals age, the PVD dendrite continues to grow and add additional 4°, as well as 5°, 6°, and irregular branches throughout adulthood [41]. Therefore, we used the 4° branch number as an indication of neuronal age. We find that dauerization indeed dramatically reduces the number of branches and delays morphological aging of PVD into late adulthood (Fig 2E). Furthermore, a hallmark of aging in diverse types of *C*. *elegans* neurons is the growth of sporadic ectopic neurites [42–44]. Remarkably, pan-neuronal dauerization reduces the prevalence of these ectopic neurites in anterior lateral microtubule (ALM) and posterior lateral microtubule (PLM), two other *C*. *elegans* neurons (Fig 2F and 2G), leading us to infer that dauerization may indeed present a cell-autonomous method to delay morphological aging in neurons.

### Endosome production is broadly suppressed in the delayed-aging state

To understand the mechanism underlying dauer-induced dendrite growth arrest, we probed various cell biological processes by examining the subcellular distribution of organelles. Numerous compartments and/or processes appear morphologically unaltered by dauerization, including the endoplasmic reticulum, Golgi, mitochondria, and autophagy (S3 Fig). PVD-specific RNA sequencing of wild-type versus dauerized neurons identified several hundred genes with altered expression in dauerized PVD, but it was not obvious how any of the changes detected would result in arrested growth (S1 Table).

**Fig 3:**
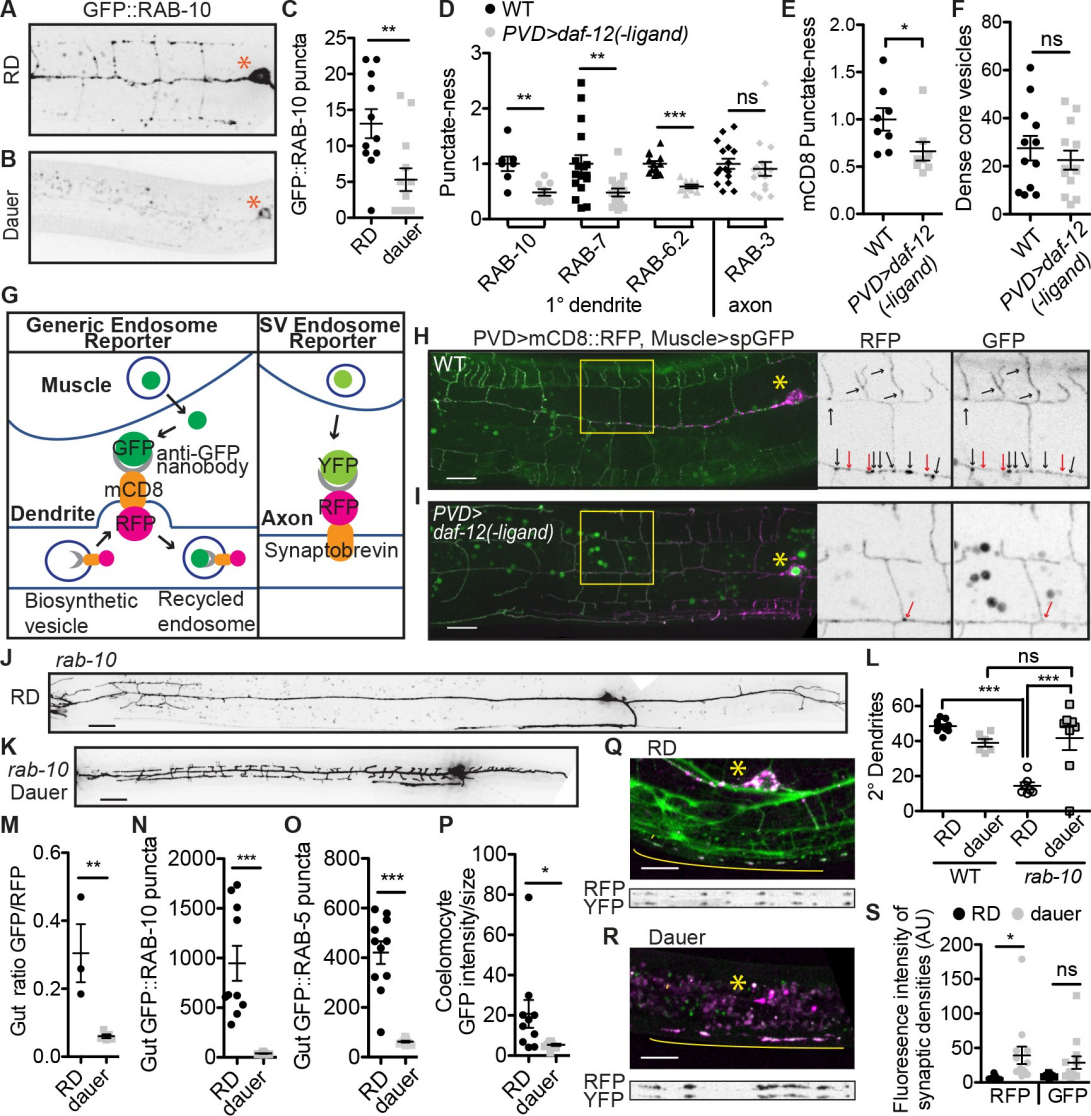
Endosome production is broadly reduced in dauerized cells. (A–C) GFP::RAB-10 localizes to vesicles throughout the dendrite in RD (panels A, C), and this localization is reduced in the dauer or dauerized dendrite (panels B, C). (D–E) Various GFP::RAB GTPases show reduced vesicular localization in dauerized dendrite, as does the generic transmembrane protein mCD8::GFP. “Punctate-ness” is the standard deviation/median intensity for each image along the proximal 1° dendrite or, for GFP::RAB-3, the axon. (F) Dense-core vesicle abundance in the proximal 1° dendrite appears unaltered in dauer. (G) Strategy for distinguishing between biosynthetic vesicles and endosomes. (H, I) The Generic Endosome Reporter shows that most vesicles outside of the PVD cell body have been recycled off the plasma membrane in RD (H), and these recycled endosomes are absent in dauerized PVD. (I) Black and red arrows in enlarged panels point to vesicles that are co-labeled or visible only in RFP channel, respectively. (J–L) The *rab-10(o)* dendrite growth defect is amended by the dauer state. (M) In dauer intestinal cells, the Generic Endosome Reporter shows reduced endosomes. (N–O) Endosome-localized GFP::RAB-10 (panel N) and GFP::RAB-5 (panel O) is reduced from vesicles in the dauer intestine compared to RD. (P) Accumulation of secreted GFP by coelomocytes is decreased in dauer. (Q–S) SV recycling is not reduced in dauer compared to RD. Grayscale images show the straightened synaptic region for the total SNB-1 (RFP) versus the endocytosed Synaptobrevin/SNB-1 (GFP). (C–F,M–P, S) ***P < 0.001, **P < 0.01, *P < 0.05, two-tailed *t* test. (L) ***P < 0.001, one-way ANOVA with Tukey post test. See also S1 Data. GFP, green fluorescent protein; GTPase, guanosine triphosphate; mCD8, mammalian cluster of differentiation 8; ns, not significant; RFP, red fluorescent protein; RD, reproductive development; SNB-1, synpatobrevin; spGFP, signal peptide green fluorescent protein; SV, synaptic vesicle; WT, wild type; YFP, yellow fluorescent protein.

We next examined RAB-10, a small GTPase required for PVD dendrite morphogenesis [45,46]. Green fluorescent protein (GFP)::RAB-10 localizes to vesicles throughout the dendrite in RD and is thought to promote vesicle exocytosis [45,46]. Strikingly, both fully dauer or dauerized PVD dendrites contain dramatically fewer GFP::RAB-10 vesicles than wild-type in RD (Fig 3A–3D, S4A Fig). Similarly, RAB-7, which localizes to late endosomes, and RAB-6.2, which localizes to recycling endosomes, both localize to vesicles throughout the dendrite in RD and show strongly reduced vesicular localization in the dauerized PVD [46,47] (Fig 3D, S4B and S4C Fig). In addition, the generic transmembrane protein mouse cluster of differentiation 8 (mCD8)::GFP exhibits reduced vesicular localization in the dauerized dendrite compared to wild type (Fig 3E). These data show that the dauerized PVD dendrite is largely devoid of vesicles in the endocytic system.

Interestingly, the abundance of dense core vesicles appears unaltered in the dauerized dendrite (Fig 3F), leading us to hypothesize that the vesicles in the PVD dendrite are predominantly recycling endosomes, and so dauerized PVD has normal production of biosynthetic secretory vesicles but reduced production of endosomes. To test this theory directly, we generated a reporter to distinguish between biosynthetic and recycled vesicles, the “Generic Endosome Reporter” (Fig 3G). In this reporter, the PVD neuron expresses the mCD8 transmembrane domain fused to red fluorescent protein (TagRFP) on the cytosolic side and GFP-binding protein (GBP), the GFP-binding nanobody, on the luminal/extracellular side. GFP is expressed and secreted from the muscle cells into the pseudocoelom so that any mCD8 molecule that has reached the plasma membrane will be both red and green, whereas biosynthetic mCD8 molecules that have not reached the plasma membrane will be red only. This reporter shows that most dendritic vesicles have indeed been recycled off the plasma membrane (Fig 3H). Similar results are observed with an endocytosis reporter made with the PVD guidance receptor dendrite morphology abnormal (DMA-1) [48] (S4F and S4G Fig). Additionally, we observe many DMA-1::GFP-containing vesicles in the PVD dendrite in RD or post dauer, but almost no DMA-1-containing vesicles in the dauer dendrite with a concomitant increase in diffuse DMA-1 along dendritic branches (S4H and S4I Fig). These data indicate that generating endosomes off the plasma membrane is a prominent feature of the PVD dendrite in RD, including endosomes containing the dendrite guidance receptor DMA-1, and that this process is suppressed in dauer.

The hypothesis that dendritic vesicles are predominantly endosomes and the known role of RAB-10 in recycling endosomes suggest that the function of RAB-10 may be to recycle endocytosed membrane proteins back to plasma membrane. Indeed, we find that the *rab-10(o)* dendrite morphogenesis defect in RD is completely suppressed in dauer (Fig 3J–3L), indicating that endosome generation is upstream of RAB-10 function and that RAB-10 is not required to deliver biosynthethic vesicles from the soma directly to the plasma membrane. Dauer’s lack of endocytosis bypasses the need for RAB-10 to return membrane proteins to the plasma membrane.

Is the reduction in endosome production specific to the PVD dendrite or a general feature of cells in dauer? In dauer intestinal cells, the generic endosome reporter shows reduced endocytosis, and GFP::RAB-10 exhibits markedly reduced punctate accumulation compared to cells in RD (Fig 3M and 3N, S4J and S4K Fig). Furthermore, Rab5/GFP::RAB-5, a ubiquitous marker for early endosomes [49], shows strongly decreased vesicular accumulation in the dauer intestine compared to RD (Fig 3O, S4L Fig). Finally, coelomocytes, which constitutively endocytose the pseudo-coelomic fluid during RD, exhibit strongly reduced accumulation of muscle-secreted GFP in dauer (Fig 3P, S4M Fig). These data indicate that reduced endocytosis is indeed a general feature of cells in dauer.

In contrast to the other fluorescently tagged RAB proteins, GFP::RAB-3, which labels SV localization, appears similar in dauerized PVD neurons compared to RD (Fig 3D). This indicates that either SV recycling is protected from the dauer-induced suppression of endocytosis or that these SVs are biosynthetic and have not been released and recycled. To distinguish between these possibilities, we constructed the “SV Endosome Reporter” (Fig 3G). In contrast to the Generic Endosome Reporter in both the PVD dendrite and intestine, the SV Endosome Reporter shows that SVs are indeed recycled in dauer (Fig 3Q–3S). In fact, we observe increased intensity of both RFP::SNB-1/Synaptobrevin and GFP::RAB-3 in dauer compared to RD in PVD (Fig 3S, S4E Fig). Similarly, endogenously tagged Synaptogyrin/SNG-1::GFP shows no decrease in vesicular localization or intensity in the ventral nerve cord in dauer compared to RD (S4M and S4N Fig). These data suggest that the specialized vesicle recycling related to synaptic function may be uniquely preserved among endocytic recycling processes in dauer.

We next attempted to suppress endocytosis in the PVD dendrite by genetic manipulations of known endocytosis effectors; however, we were unable to reduce the dendritic prevalence of DMA-1::GFP endosomes with a temperature-sensitive allele of dynamin/*dyn-1* [49] (S5A Fig). Furthermore, loss-of-function of the clathrin adaptor AP-1μ/*unc-101*, the clathrin- and dynamin-independent endocytosis mediator Arf6/*arf-6*, and an *unc-101; arf-6; rme-1* triple mutant [49] all showed no obvious reduction in the prevalence of dendritic GFP::RAB-10 vesicles compared to wild type (S5B Fig).

### Endosomes control the localization and activity of actin regulator TIAM-1 to effect PVD dendrite morphogenesis versus growth arrest

**Fig 4:**
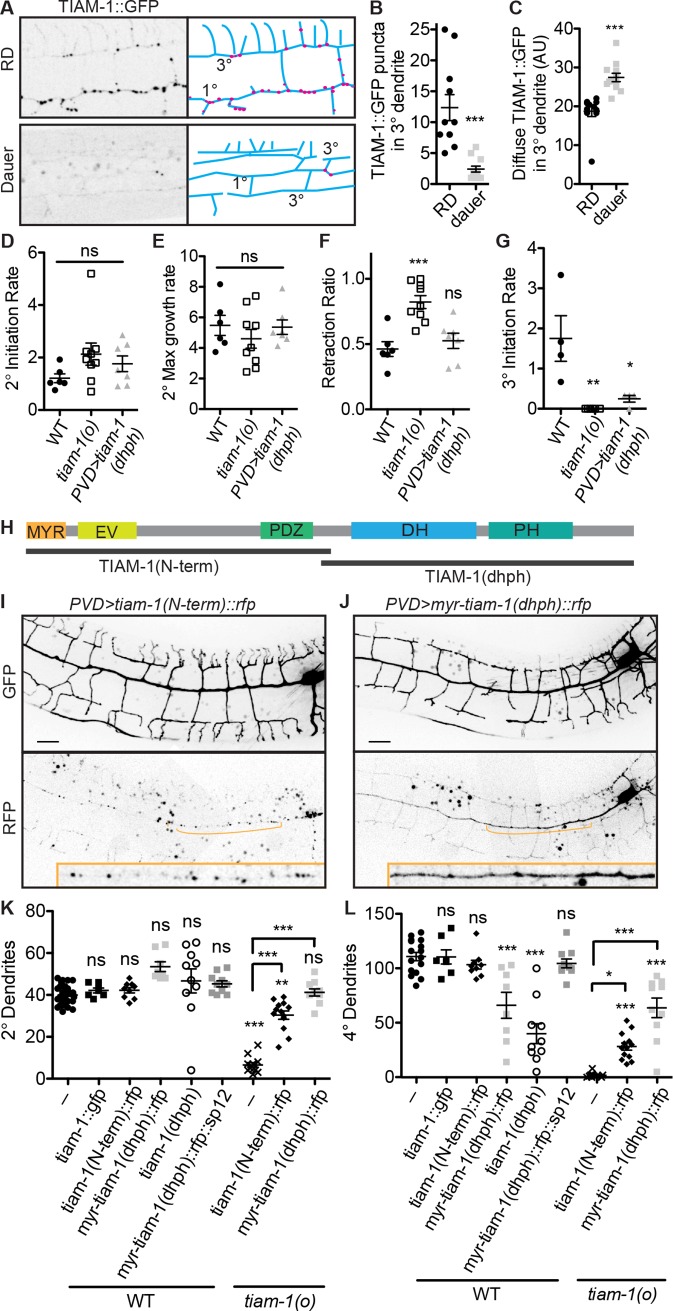
Localization of the Rac GEF TIAM-1 to the dendrites promotes dendrite stabilization, whereas localization to vesicles allows growth. (A–C) The Rac GEF TIAM-1 exhibits both diffuse and vesicular localization in RD but is predominantly diffuse in dauer. ***P < 0.001, two-tailed *t* test. (D–F) Quantification of dendrite growth parameters from movies of wild-type *tiam-1(o)* mutant, and *tiam-1(dhph)* overexpression animals (see 4H and example S1–S3 Movies). The *tiam-1(o)* mutant exhibits similar dendrite extension frequency (D) and growth rate (E) but an increased frequency of retraction (F) compared to wild type. (G) Overexpression of the TIAM-1(dhph) fragment causes reduced frequency of 3° initiation. Each data point was calculated from a 2- to 3-hour movie of a single growing dendritic arbor as described in Materials and methods. *P < 0.05, **P < 0.01, **P < 0.001, one-way ANOVA with Tukey post test. (H) Schematic diagram of TIAM-1 with truncated expression constructs indicated. (I, K–L) TIAM-1 N-terminal truncation exhibits vesicular localization (panel I, bottom) and does not affect dendrite morphology in the wild-type background (panel I, top, panels K–L). (J, K–L) TIAM-1 C-terminal truncation exhibits diffuse localization (J, bottom panel) and induces a dauer-like dendrite morphology in a wild-type background, but tethering this fragment to the ER by fusing it to SP12 abrogates this gain-of-function phenotype (panel J, top, panels K–L). Both TIAM-1 fragments can partially rescue the *tiam-1(o)* mutant dendrite morphology defect (K–L). *P < 0.05, **P < 0.01, **P < 0.001, one-way ANOVA with Tukey post test, significance is in relation to wild type except where otherwise noted. See also S1 Data. DH, Dbl homology; ER, endoplasmic reticulum; EV, Ena/VASP homology domain; GEF, guanine nucleotide exchange factor; GFP, green fluorescent protein; MYR, myristoylation; ns, not significant; RD, reproductive development; SP12, secretory protein 12; TIAM-1, T cell lymphoma invasion and metastasis 1; WT, wild type.

How does suppressing general endosome production lead to arrested dendrite development and aging? Our previous work showed that PVD dendrite morphogenesis is mainly driven by the actin cytoskeleton through the activation of regulators such as WASP-family verprolin homologous protein 1 (WAVE) Regulatory Complex and TIAM-1 [50]. TIAM-1 is a putative Rac GEF and scaffold for numerous actin regulators that is important for neuron morphogenesis from worms to mammals [51]. *tiam-1(o)* mutants have a severely stunted PVD dendritic arbor with almost no 2° or 4° branches, and TIAM-1::GFP exhibits a vesicular localization in the PVD dendrite [50,52] (Fig 4A). Examining TIAM-1 localization in dauer or dauzerized PVD, we observe a loss of vesicular TIAM-1::GFP with a concomitant increase in diffuse cytosolic TIAM-1::GFP (Fig 4A–4C, S6A–S6D Fig). We asked whether this alteration in TIAM-1 localization might contribute to the dauer dendrite growth arrest and, if so, how.

First, to better understand the function of TIAM-1, we examined dendrite growth parameters using live imaging of *tiam-1(o)* versus wild-type worms at the RD developmental stage in which 2° and 3° PVD branches are forming. Both neurite outgrowth frequency and growth speed in the *tiam-1(o)* mutant are indistinguishable from that of wild type, whereas neurite retraction is increased in the *tiam-1(o)* mutant, resulting in fewer 3° dendrite growths (Fig 4D–4G, S1 Movie and S2 Movie). This suggests that the primary function of TIAM-1 in these dendrites is to promote stability but not outgrowth. Though it has often been implicated in migration, mammalian Tiam1 has likewise been shown to support cell adhesion in some contexts [53].

Next, to understand the function of vesicular versus diffusely localized TIAM-1, we performed structure-function analyses. We divided TIAM-1 into the C-terminal portion, “TIAM-1(dhph),” which contains Rac GEF Dbl homology (DH) and pleckstrin homology (PH) domains, and the N-terminal portion, “TIAM-1(N-term),” which is required for membrane localization and numerous protein interactions and is auto-inhibitory for the GEF activity in vitro [51] (Fig 4H). TIAM-1(N-term)::RFP localizes to vesicles throughout the PVD dendrite, like full-length TIAM-1 in RD, whereas TIAM-1(dhph)::RFP exhibits diffuse localization, like full-length TIAM-1 in dauer (Fig 4I and 4J). Remarkably, overexpression of the TIAM-1(dhph) fragment in the wild-type RD background results in a dendrite with fewer 4° branches, similar to the dauerized dendrite morphology (Fig 4J, 4K and 4L). This is not simply a dominant-negative, or inhibitory, function of TIAM-1(dhph) fragment, as this fragment is sufficient to partially rescue dendrite morphology of the *tiam-1(o)* mutant, again to a dauer-like morphology with wild-type 2°s but few 4°s (Fig 4K and 4L). Furthermore, tethering the TIAM-1(dhph) fragment to the endoplasmic reticulum completely abolishes its ability to inhibit 4° branch growth, showing that the diffuse localization likely representing cytosolic TIAM-1 is essential for this function (Fig 4K and 4L). It is therefore likely that diffuse TIAM-1(dhph) in the 3° branches acts to inhibit 4° growth. Examining dendrite growth parameters in the TIAM-1(dhph) (nontethered) strain, we find that 3° dendrites are initiated at a reduced frequency compared to wild type (Fig 4G) (S3 Movie). Together with our analyses of the *tiam-1(o)* mutant, these data indicate that diffusely localized TIAM-1 promotes dendrite stabilization but inhibits 4° branch growth via the TIAM-1(dhph) domain.

**Fig 5:**
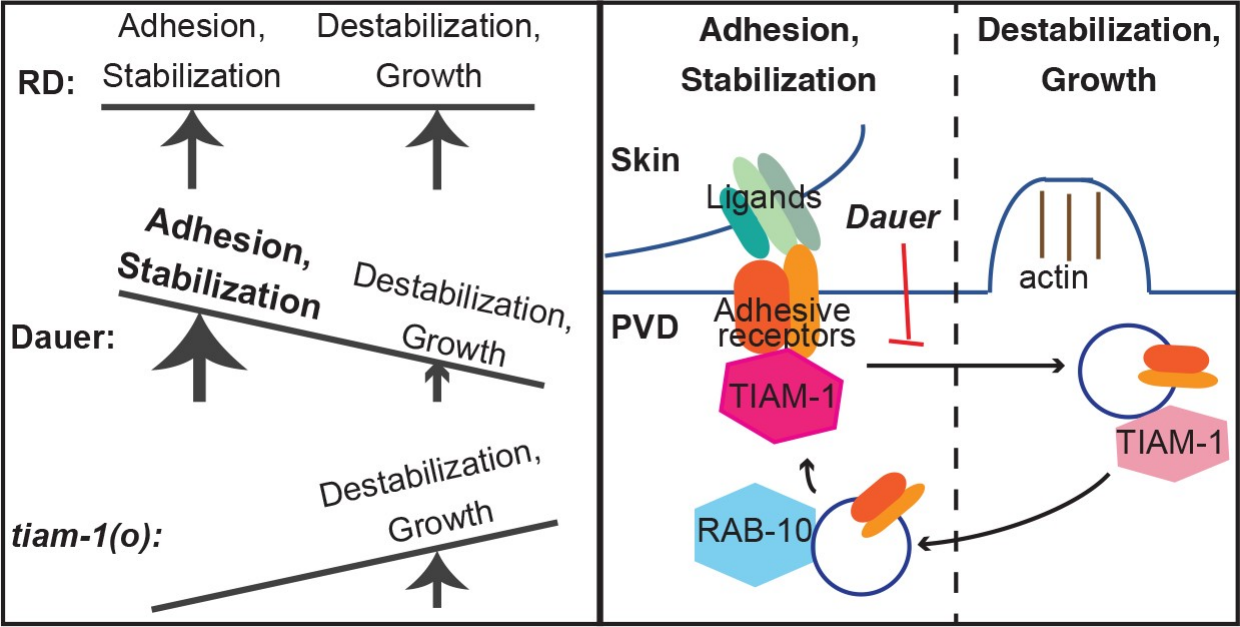
Summary model. (Left) In RD, dendrite adhesion and stabilization are balanced with destabilization and growth to promote dendrite morphogenesis, whereas in dauer, the balance is tipped to favor stabilization, preventing growth. The *tiam-1(o)* mutant lacks stabilization, so neurite growths are retracted. (Right) This balance between stabilization and growth is regulated by endosome generation, which is constitutive in RD and ceased in dauer. Diffuse TIAM-1 promotes neurite stabilization, likely through interactions with guidance receptors at the plasma membrane, and TIAM-1 localization to endosomes allows, and perhaps promotes, higher-order branch growth. RD, reproductive development; TIAM-1, T cell lymphoma invasion and metastasis 1.

In contrast to TIAM-1(dhph), the endosome-localized TIAM-1(N-term) has no effect on dendrite morphology in the wild-type background (Fig 4I, 4K and 4L). Interestingly, it does provide partial rescue of dendrite morphology in the *tiam-1(o)* mutant (Fig 4K and 4L), indicating that TIAM-1 has at least 2 separable functional domains that promote dendrite morphogenesis. The vesicle-localized TIAM-1 is therefore likely active, perhaps as scaffolding for a signaling pathway, rather than just sequestered and inactivated. Taken together, we propose that DAF-12 cell-autonomously regulates the overall abundance of endosomes, which adjusts the balance of vesicular and cytosolic TIAM-1 (Fig 5). In dauer-like neurons, the scarcity of endosomes increases the cytosolic TIAM-1 and causes overstabilization of 3˚ branches and inhibition of new branch formation.

## Discussion

Here, we have elucidated a hormone-regulated mechanism that can cell-autonomously delay neuronal morphological development and aging. In addition, we propose a surprising modification to the current model for how neurite outgrowth is effected (see additional discussion to follow). In summary of the first point, cells in the slowed-aging state broadly suppress endosome production, though SV cycling is uniquely preserved. In the PVD dendrite, endosomes localize the actin regulator TIAM-1 to permit dendrite growth in RD or stall it in dauer (Fig 5). Future experiments will address whether additional aspects of neuronal aging can likewise be delayed by dauerization (DAF-12(−ligand)) and, if so, how.

Endocytosis is fundamental across cell types. While it is perhaps most active, or at least most studied, in proliferating cells, endocytosis and endosomes perform myriad roles in the homeostasis and physiology of terminally differentiated cells [4]. That dauerization appears to present a physiological mechanism that broadly suppresses endocytosis is therefore somewhat surprising. It was not obvious from our cell-specific sequencing what transcriptional changes induced by *daf-12(−ligand)* would lead to suppressed endosome generation, and we were unable to inhibit PVD dendritic endosomes through manipulation of known endocytosis effectors (S5 Fig). There are multiple parallel pathways that mediate constitutive endocytosis [3, 49], so depleting one or several of these pathways leaves the possibility that others will compensate.

Animals in dauer diapause do not eat, so many of the cellular changes associated with dauer diapause, such as decreased transcription, altered metabolism, and increased autophagy, are thought to be energy-conservation mechanisms [25]. Broadly suppressing constitutive endocytosis could likewise contribute to energy conservation in dauer, and it may be a factor in the extended RD lifespan observed in the *daf-12(rh273)* mutant [38]. Still, SV recycling is maintained in the dauer state. This is consistent with the fact that animals continue to behave throughout dauer diapause—they respond to smells and touch, and they locomote with the appearance of seeking a more favorable environment [54, 55]. We therefore speculate that this phenomenon of suppressing constitutive endocytosis allows *C*. *elegans* to not only halt the growth and other age-related programs supported by most endosome recycling but also conserve energy while maintaining neurotransmission, and thus behaviors, during dauer diapause.

In neuron morphogenesis and remodeling, it is generally thought that the mechanisms producing the actin-mediated filopodial and lamellipodial protrusions as well as those stabilizing the growth cone are both forces that promote growth cone extension [56]. By examining dendrite outgrowth in RD versus dendrite growth arrest in dauer, our data support a modification to this model in which the amount of stabilization must be balanced: if there is not enough stabilization, neurites fail to grow because protrusions are retracted, but excessive stabilization antagonizes outgrowth (Fig 5). Down-regulation of constitutive endosome generation presents an endogenous mechanism to tip the balance toward stabilization in dauer, likely due to TIAM-1 working with adhesive receptors at the plasma membrane. Consistent with the model that excessive stabilization antagonizes outgrowth, there is an increase in the level of the adhesive guidance receptor DMA-1 on the plasma membrane in dauer relative to RD (S4H–S4I Fig). In our previous work we found that DMA-1 is required for neurite stabilization, and overexpression of DMA-1 in RD causes a reduction, rather than an increase, in the number of 4° dendrites [48,57]. We propose that the constitutive endosome generation of RD, which could locally remove both TIAM-1 and adhesive receptors from the 3° dendrite, causes local destabilization to allow growth. This could involve swapping a stabilizing branched F-actin network for a growing filopodial actin bundle. Interestingly, although TIAM-1 has been shown to promote PVD morphogenesis by regulating actin, this may not involve its Rac GEF activity—a single amino-acid substitution can abrogate TIAM-1 GEF activity in vitro, and making this mutation in *tiam-1 in vivo* has no effect on PVD morphology [50,52]. How branch growth is initiated along an existing neurite is not well understood, and we speculate that reducing neurite stabilization locally may be an important early step in that process.

## Materials and methods

### *C*. *elegans* strains and maintenance

*C*. *elegans* strains were cultured on *Escherichia coli* OP50 as described by Brenner [58]. Data for worms in RD were collected from L4-stage animals unless otherwise noted. For Fig 1B and 1D, S1, S4M and S4N Figs (“WT dauers”), dauer animals were isolated from starved populations using 1% SDS (Sigma-Aldrich, St. Louis, MO) wash. All other dauer animals were prepared using the temperature-sensitive *daf-2(e1370)* genetic background, which grows though RD at 16°C and into dauer at 25°C. Dauers were grown from eggs at 25°C for 5 days, and they were compared against RD worms of the same strain grown at 16°C to the L4 stage. In all transgenes expressing *daf-12* variants, *daf-12* was translationally tagged with gfpnovo2, allowing verification of expression and nuclear localization of the construct. Several strains were provided by the Caenorhabditis Genetics Center (CGC), which is funded by NIH Office of Research Infrastructure Programs (P40 OD010440). The following strains were used in this study: TV15921 *wyIs594(ser2prom3*::*myr-gfp*, *Podr-1*::*rfp)*, TV20384 *daf-2(e1370); wyIs594*, TV22406 *din-1(dh149); daf-2(e1370); wyIs594*, TV23318 *din-1(dh149); daf-2(e1370); wyIs594; wyEx9453(ser2prom3*::*din-1*, *Punc-122*::*rfp)*, TV22125 *wyIs594; daf-12(rh61rh411)*, TV23204 *wyIs594; daf-12(rh273)*, TV22536 *wyIs594; wyEx9238(ser2prom3*::*daf-12a*::*gfpnovo2*, *Pmyo-2*::*gfp)*, TV22537 *wyIs594; wyEx9239(ser2prom3*::*daf-12a*::*gfpnovo2*, *Pmyo-2*::*gfp)*, TV22833 *wyIs840(ser2prom3*::*daf-12a(rh273)*::*gfpnovo2*, *Pmyo-2*::*gfp); wyIs592(ser2prom3*::*myr-gfp)*, TV22534 *wyIs594; wyEx9236(Phlh-1*::*daf-12a(rh273)*::*gfpnovo2*, *Pmyo-2*::*gfp*, TV11461 *wyEx4684(Pmec-17*::*mcherry*, *Podr-1*::*rfp)*, TV24585 *wyIs1178(Prgef-1*::*daf-12a(rh273)*::*gfpnovo2*, *ser2prom3*::*myr-gfp); wyEx4684*, TV23226 *wyIs594; daf-12(rh61rh411); wyEx9413(ser2prom3*::*EnR*::*daf-12(DNA-binding-domain)*::*gfpnovo2*, *Punc-122*::*rfp)*, TV23225 *wyIs594; daf-12(rh61rh411); wyEx9412(ser2prom3*::*VP64*::*daf-12(DNA-binding-domain)*::*gfpnovo2*, *Punc-122*::*rfp)*, TV21537 *wyIs50025(ser2prom3*::*sp12*::*gfp*, *ser2prom3*::*mcherry*, *Podr-1*::*gfp)*, TV23485 *wyIs840; wyIs50025*, NK1351 *qyIs296(ser2prom3*::*mans*::*gfp*, *ser2prom3*::*mcherry*, *unc-119(+))*, TV23069 *wyIs840; qyIs296*, TV24940 *wyIs235006(ser2prom3*::*gfp*::*tomm-20))*, TV23354 *wyIs840; wyIs235006*, MAH242 *sqIs24(Prgef-1*::*gfp*::*lgg-1*, *Punc-122*::*rfp)*, TV23205 *wyIs840; sqIs24*, TV23488 *daf-2(e1370); wyEx7701(Pdes-2*::*gfp*::*rab-10*, *ser2prom3*::*myr-mcherry*, *Podr-1*::*rfp)*, TV23337 *wyIs840; wyEx7701*, TV17379 *qyIs369; wyEx7137(ser2prom3*::*mcherry*::*rab-7*, *Podr-1*::*gfp*, TV23553 *wyIs840; wyEx7137*, TV23494 *wyEx9514(ser2prom3*::*gfp*::*rab-6*.*2*, *Punc-122*::*rfp)*, TV24096 *wyIs840; wyEx9514*, TV12922 *wyEx5216(Pdes-2*::*gfp*::*rab-3*, *ser2prom3*::*myr-mcherry*, *Podr-1*::*rfp)*, TV23481 *wyIs840; wyEx5216*, TV17207 *wyIs581(ser2prom3*::*myr-mcherry*, *Podr-1*::*gfp)*, *wyEx7069(ser2prom3*::*mcd8*::*gfp*, *Podr-1*::*rfp)*, TV23567 *wyIs856(ser2prom3*::*nlp-12*::*venus*, *Podr-1*::*rfp)*, TV23258 *wyIs840; wyIs856*, TV23698 *smg-1(r861); wyEx9576(Pmyo-3*::*sp-gfp*, *ser2prom3*::*sp-gbp(gfp nanobody)*::*mcd8*::*tagrfp*, *Pmyo-2*::*rfp)*, TV24551 *rab-10(ok1494); daf-2(e1370); wyIs594*, TV24540 *daf-2(e1370); wyEx9853(Pmyo-3*::*sp-gfp*, *Pvha-6*::*gbp*::*mcd8*::*tagrfp*, *Podr-1*::*rfp)*, TV24432 *daf-2(e1370)*, *pwIs206(Pvha-6*::*gfp*::*rab-10*, *unc-119+)*, *daf-2(e1370); arIs37(Pmyo-3*::*sp-gfp); wyIs93(Pglr-3*::*mcherry*::*rab-3*, *Pglr-3*::*glr-1*::*gfp*, *Punc-122*::*rfp)*, TV24912 *daf-2(31370); wyEx9934(ser2prom3*::*snb-1*::*tagrfp*::*gbp*, *Pmyo-3*::*sp-gfp*, *Podr-1*::*rfp)*, TV24514 *dma-1(tm5159); wyEx1240(ser2prom3*::*4xgcn4*::*dma-1*::*tagrfp*, *Pmyo-3*::*scFV(gcn4-nanobody)*::*superfoldGFP*, *Podr-1*::*rfp)*, TV21000 *daf-2(e1370); wyIs740*, TV22165 *wyIs1139(ser2prom3*::*tiam-1*::*gfp*, *Pmyo-2*::*mCherry)*, TV17428 *tiam-1(tm1556); wyIs594*, TV24791 *wyIs592; wyEx9908(ser2prom3*::*tiam-1(dhph)*, *Podr-1*::*gfp*, TV24877 *wyIs592; wyEx9929(ser2prom3*::*myr-tiam-1(dhph)*::*tagrfp*, *Podr-1*::*gfp)*, TV24979 *tiam-1(tm1556); wyIs592; wyEx9929*, TV24880 *wyIs592; wyEx9930(ser2prom3*::*myr-tiam-1*::*dhph)*::*tagrfp*::*sp12*, *Podr-1*::*gfp)*, TV20605 *dyn-1(wy1150); wyIs740*, EG9408 *sng-1(ox706)*, TV24431 *daf-2(e1370); pwIs72(Pvha-6*::*gfp*::*rab-5)*.

### Molecular biology and transgenes

Expression vectors were made in the pSM vector, a derivative of pPD49.26, or pPD117.01 (Addgene, Watertown, MA) using standard techniques. Transgenes expressed from extrachromosomal or integrated arrays were generated using standard gonad transformation by injection [59]. *tiam-1* truncation constructs were as follows: pCER255(ser2prom3::myr-tiam-1(dhph)(aa519-889)::tagrfp); pCER258(ser2prom3::tiam-1N-term(aa1-540)::tagrfp); pCER261(ser2prom3::tiam-1(dhph)(aa519-889); and pCER269(ser2prom3::myr-tiam-1(dhph)(aa519-889)::tagrfp::sp12).

### Confocal imaging and fluorescence microscopy

Visual inspections of fluorescence were performed using a Zeiss Axioplan 2 microscope with a 63×/1.4NA objective and Chroma HQ filter sets for GFP, YFP, and RFP. Animals were immobilized in 10 mM levamisole (Sigma, St Louis, MO) in M9 buffer. Images were acquired with either a Zeiss LSM710 confocal microscope using a Plan Apochromat 63×/1.4 objective or an inverted Zeiss Azio Observer Z1 spinning disk confocal microscope with a 63×/1.4NA or 40× objective attached to a QuantEM:512SC camera. Images were analyzed in ImageJ. Light artifacts due to scatter from the spinning disk were removed from images of PVD morphology with ImageJ. Puncta were quantified from images using either “find maxima” (Figs 3C, 3F, 3N and 4B) or with “analyze particles” (Fig 3O and 3S, S6C Fig), or by eye on the compound microscope (S3E and S3F Fig). For movies of dendrite outgrowth, animals were immobilized in 5 mM levamisole in M9 buffer, and confocal stacks were acquired every 3 minutes for 2 to 3 hours. Quantifications were performed as follows: extension frequency: 2°s initiated/1° dendrite length visible/hour; growth rate: longest 2° growth event (in pixels)/3 minutes; frequency of retraction: 2°s initiated/2°s retracted; and 3° initiation rate: 3°s with proper orientation and location initiated from 2°s/hour.

### FACS isolation of dissociated cells

Synchronized L4 worms with GFP-labeled neurons (wyIs592) were prepared for cell isolation as previously described [60]. Cells were filtered using 5 μm cell strainers (Corning, Corning, NY) and resuspended in AccuMax (Sigma) on ice. Cell viability dyes (Hoescht 33342 Ready Flow Reagent [Thermo Fisher Scientific, Waltham, MA]) and Propidium Iodide Ready Flow Reagent (Thermo Fisher Scientific) were added to the cells according to manufacturer directions. The filtered cells were sorted using a BD FACSAria Fusion with a 100 μm nozzle equipped with a blue laser for GFP detection (488 nm, 525/50 filter with B525 detector), UV laser for Hoescht detection (355 nm, 515/30 filter with U515 detector), and yellow laser for propidium iodide (PI) detection (561 nm, 670/30 with Y660 detector). Negative GFP gates were established using N2 worms that were prepared alongside the experimental samples (wyIs592 and wyIs592;wyIs840). Events that were positive for Hoescht signal and negative for PI signal were gated for GFP and sorted into 1.5 mL tubes containing RNAlater (Thermo Fisher Scientific, Waltham, MA) and kept on ice. Approximately 12,000 to 70,000 positive events were collected for each test group (wyIs592 GFP+, wyIs592 GFP⁻, wyIs592;wyIs840 GFP+, and wyIs592;wyIs840 GFP⁻). Sorted cells were spun down at 10,000 RPM for 10 minutes at 4°C, and RNA was extracted using NucleoSpin RNA XS (Macherey-Nagel, Duren, Germany) and analyzed for integrity using an Agilent Bioanalyzer 2100. Samples with RIN values greater than 8 were converted to cDNA using the SMART-seq version 4 Ultra low input kit (Takara Bio USA, Mountain View, CA). Libraries were prepared using the Nextera XT kit (Illumina, San Diego, CA) as per the SMART-seq version 4 protocol. The resultant libraries (3 biological repeats per condition) were pooled and sequenced on the Illumina HiSeq 4000 platform at the Stanford Genome Sequencing Service Center.

### RNA sequencing analysis

Sequencing samples were initially analyzed by FastQC to assess sequencing quality and sequencing bias. TrimGalore (0.5.0), a tool that combines Cutadapt and FastQC, was used to remove low-quality base calls and the Illumina adapter from the 3′ end of reads. The reads for each sample were mapped to the reference genome (ce10) using TopHat (version 2.1.1), and expression levels and statistical significance of observed changes were analyzed using the complete Cufflinks pipeline (version 2.2.1). Lists of differentially expressed genes were analyzed for GO term enrichment using DAVID (version 6.8).

## Supporting information

S1 Data Excel spreadsheet containing, in separate sheets, the underlying numerical data and statistical analysis for Fig panels 1D, 2A, 2D, 2E, 2F, 2G, 3C, 3D, 3E, 3F, 3L, 3M, 3N, 3O, 3P, 3S, 4B, 4C, 4D, 4E, 4F, 4G, 4K, 4L, S1, S2C, S3C, S3G, S3H, S4E, S4I, S4O, S6C, and S6D. (XLSX)

S1 Fig Other endocrine signaling components involved in the organismal dauer state decision do not affect PVD dendrite growth arrest in dauer. Shown are loss-of-function mutants in *daf-2/insulin like-receptor*, *daf-3/SMAD*, *daf-7/TGFβ*, and *daf-16/FOXO*. The *daf-3* and *daf-16* mutants are resistant to entering dauer, like the *din-1* mutant, so either the *daf-2* or the *daf-7* mutation was added to their genetic background to enable dauer entry. Data for wild type are the same as that shown in Fig 1.
(TIF)

S2 Fig Dauer-like dendrite growth arrest is induced by transcriptional repressive function of DAF-12. (A–B) *daf-12(o)* animals expressing the DAF-12 DBD attached to a trans-repressive (EnR) (panel A) or trans-activating (VP64) (panel B) domain driven from a PVD-specific promoter. (C) The transcriptionally repressive DAF-12 chimera cell-autonomously reduces dendrite growth in RD. ***P < 0.001, one-way ANOVA with Tukey post test. DBD, DNA-binding domain.
(TIF)

S3 Fig Various facets of PVD cell biology appear unaltered by dauerization. Endoplasmic reticulum morphology (A–C), Golgi morphology (D), mitochondria (E–G), and autophagy (H) all appear similar in wild type versus dauerized (*PVD>daf-12(−ligand)*) PVD. (A) Representative image of endoplasmic reticulum morphology in a wild-type day 1 adult: it fills the primary dendrite and a (seemingly arbitrary) subset of secondary and tertiary branches [1]. (D) Representative examples of the Golgi: it localizes exclusively, or nearly so, to the cell body and forms many small stacks. (E–F) Images of PVD morphology (PVD>myr-mCherry, top) and mitochondria (PVD>tomm-20::gfp, bottom), with blue arrows pointing to the locations of the dendritic mitochondria in each image. The cell body (blue asterisk) and axon (blue line) contain many mitochondria. In the PVD dendrite, there is no obvious correlation between mitochondria localization and dendritic branching. Scale = 50 um (A–B, E–F) or 5 um (D). (C, G–H) ***P < 0.001, two-tailed *t* test. 1. Liu X, Guo X, Niu L, Li X, Sun F, Hu J et al. Atlastin-1 regulates morphology and function of endoplasmic reticulum in dendrites. Nat Commun. 2019;10: 568.
(TIF)

S4 Fig Additional characterization of the dauer-induced endocytosis block. (A–C) Localization of RAB-10, RAB-7, and RAB-6.2 is less vesicular and more diffuse in dauerized dendrite compared to wild type. (D–E) Localization of RAB-3 is similar, but with increased accumulation, in dauerized versus wild-type PVD. **P < 0.01, two-tailed *t* test. (F) Strategy for constructing the DMA-1 guidance receptor endosome reporter. DMA-1 is fused to RFP on the cytosolic side and the GCN4 peptide epitope on the extracellular side. GFP fused to anti-GCN4 nanobody is secreted from the muscle. (G) Most DMA-1-containing vesicles in the PVD dendrite have been recycled off the plasma membrane. (H–I) DMA-1 exhibits both diffuse and endosomal localization in RD but is predominantly diffuse in dauer. In the *rab-10* mutant, there is little endosomal DMA-1 in RD but much more diffuse DMA-1 in dauer, consistent with the model that RAB-10 is required to recycle endosomes to the plasma membrane and that endosome production is shut down in dauer. ***P < 0.001, one-way ANOVA with Tukey post test. (J–L) Example images of the intestinal and coelomocyte endocytosis reporters quantified in Fig 3O–3Q. The generic endosome reporter (J) expressed in the intestinal cells shows vesicular and tubular structures, many of which are co-labeled with endocytosed GFP. These tubular structures are characteristic of the recycling endosome compartment in these cells. In dauer, mCD8::RFP shows vesicular accumulations that are not co-labeled with GFP. (K) In RD, GFP::RAB-10 localizes to vesicles throughout the intestine, and these puncta are rarely observed in dauer, either when the imaged is processed in the same way as the RD image (left) or when the intensity is increased (right). (L) In RD, GFP::RAB-5 localizes to vesicles throughout the intestine, and vesicular localization is decreased in dauer. (M) Coelomocytes accumulate sGFP into endosomes in RD, and this accumulation is reduced in dauer. (N, O) Endogenously tagged Synaptogyrin/SNG-1::GFP shows punctate localization along the ventral nerve cord, and neither the punctate (vesicular) localization nor the intensity is decreased in dauer compared to RD. Scale = 10 um. sGFP, muscle-secreted GFP.
(TIF)

S5 Fig Reduced function of endocytosis effectors does not deplete endosomes from PVD dendrite. (A) Examples of DMA-1::GFP in the PVD anterior primary dendrite and cell body in the dynamin *dyn-1(wy1150)* mutant. This CRISPR-generated allele produces the same temperature-sensitive lesion as *Drosophila shibire(ts1)*, at conserved reside G273D [1]. “Permissive temperature” worms were grown at 20°C, whereas “restrictive temperature” worms were shifted from 20°C to 30°C as early L4s for 5 hours prior to imaging. Note that there is no obvious reduction in the number of DMA-1::GFP-labeled vesicles along the primary dendrite at the restrictive temperature. There appears to be an increase in DMA-1::GFP intensity in the cell body at the restrictive temperature, consistent with the model in which Dynamin/DYN-1 is required to generate transport vesicles from the Golgi. (B) Loss-of-function alleles of known mediators of endocytosis and/or endosome recycling cause no obvious reduction in the number of GFP::RAB-10-labeled vesicles in the PVD dendrite. Endocytosis regulators examined are as follows: clathrin-independent endocytosis effector Arf6—*arf-6(tm1447)* (a deletion causing a putative null), clathrin adaptor AP-1μ—*unc-101(wy50042)* (G474A mutation relative to isoform a.2, causing W158Stop), and EpsI5-homology domain (EHD) protein—*rme-1(b1045)* (a deletion causing a putative null) [2]. 1. van der Bliek AM, Meyerowitz EM. Dynamin-like protein encoded by the *drosophila shibire* gene associated with vesicular traffic. Nature. 1991;351:411–414. 2. Grant B, Zhang Y, Paupard MC, Hall DH, Hirsh D, Lin SX. Evidence that RME-1, a conserved *C*. *elegans* EH-domain protein, functions in endocytic recycling. Nat Cell Biol. 2001;3: 573–579.
(TIF)

S6 Fig TIAM-1 localization is shifted from vesicular to diffuse in dauerized PVD. In wild-type RD, GFP::TIAM-1 localizes to vesicles throughout the dendrite (A, C), and it is less vesicular and more diffuse in dauerized (*PVD>daf-12(−ligand)*) PVD (B,D). Scale = 10 um. ***P < 0.001, two-tailed *t* test.
(TIF)

S1 Table RNA sequencing of wild type versus dauerized PVD neurons. (XLSX)

S1 Movie PVD dendrite outgrowth in wild-type animal. Movie shows maximum projection from image stacks acquired every 3 minutes for 3 hours and are played at 2 frames per second. Ventral is down; anterior is left.
(AVI)

S2 Movie PVD dendrite outgrowth in *tiam-1(o)* mutant animals. PVD development is shown from 2 animals laying side by side. Movie acquired as in S1 Movie.
(AVI)

S3 Movie PVD dendrite outgrowth in TIAM-1(dhph) over-expression animal. Two animals are shown, but PVD is visible only in the lower worm. Movie acquired as in S1 Movie.
(AVI)

## Abbreviations

ALM: anterior lateral microtubule
CGC: Caenorhabditis Genetics Center
DAF: abnormal DAuer Formation
DH: Dbl homology
DIN: daf-12 interacting protein
DMA: dendrite morphology abnormal
FOXO: forkhead box O
GBP: GFP-binding protein
GEF: guanine nucleotide exchange factor
GFP: green fluorescent protein
GPCR: G-Protein Coupled Receptor
GTP: guanosine triphosphate
mCD: mouse cluster of differentiation
myr: myristoylation
NHR: nuclear hormone receptor
PH: pleckstrin homology
PI: propidium iodide
PLM: posterior lateral microtubule
RD: reproductive development
RFP: red fluorescent protein
SNB: synaptobrevin
SNG: synaptogyrin
SP12: secretory protein 12
SPEN: split ends
spGFP: signal peptide green fluorescent protein
SV: synaptic vesicle
TIAM: T cell lymphoma invasion and metastasis
WAVE: WASP-family verprolin homologous protein
