# Supplementary figures and images for: A hormone receptor pathway cell-autonomously delays neuron morphological aging by suppressing endocytosis
Source: PLoS Biol. 2019 Oct 7;17(10):e3000452. doi: 10.1371/journal.pbio.3000452 (PMC6797217; doi:10.1371/journal.pbio.3000452)

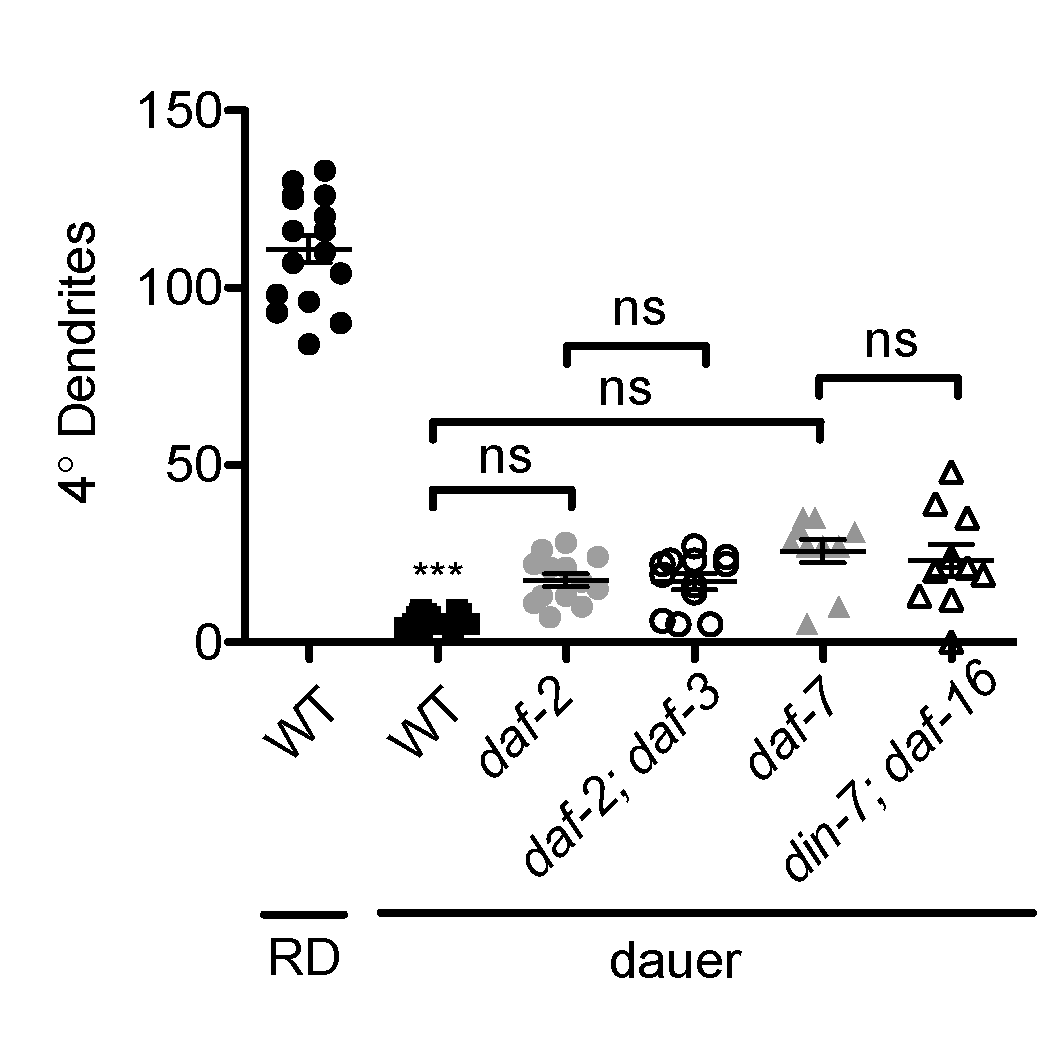

Supplement: S1 Fig — Shown are loss-of-function mutants in daf-2/insulin like-receptor, daf-3/SMAD, daf-7/TGFβ, and daf-16/FOXO. The daf-3 and daf-16 mutants are resistant to entering dauer, like the din-1 mutant, so either the daf-2 or the daf-7 mutation was added to their genetic background to enable dauer entry. Data for wild type are the same as that shown in Fig 1. (TIF) [file pbio.3000452.s002.tif]

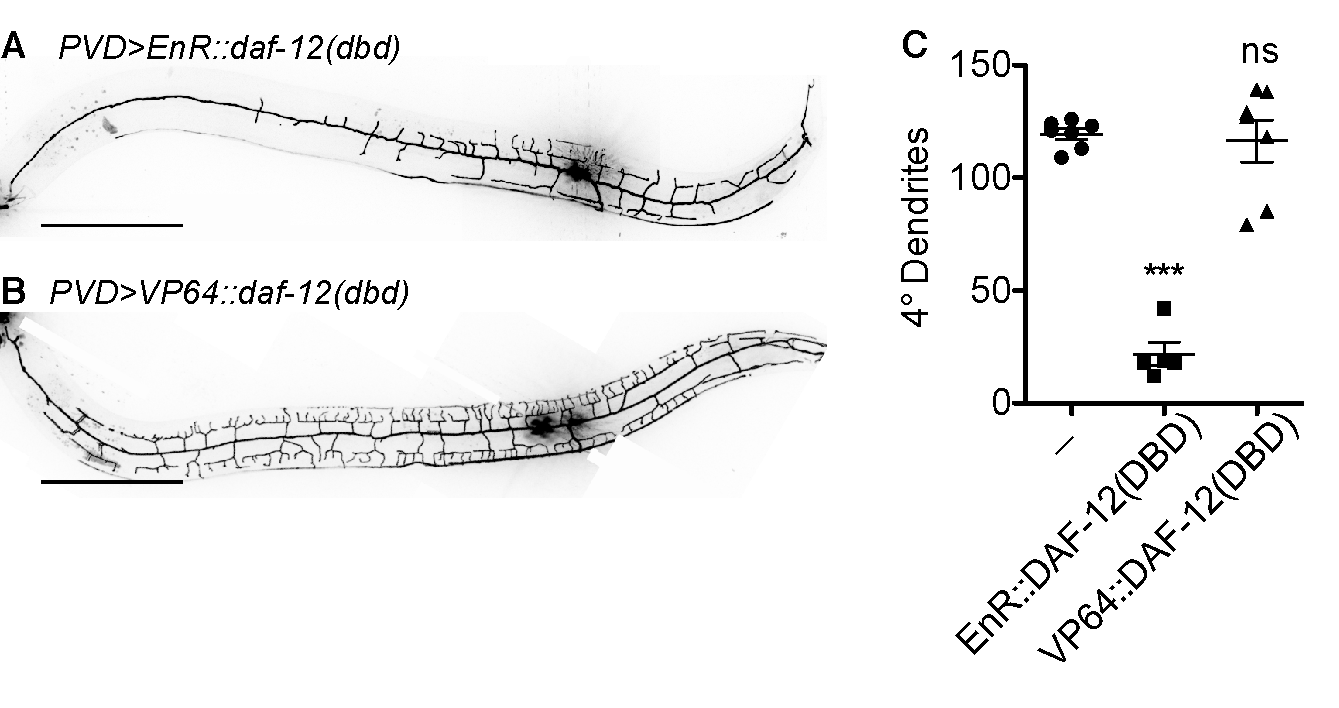

Supplement: S2 Fig — (A–B) daf-12(o) animals expressing the DAF-12 DBD attached to a trans-repressive (EnR) (panel A) or trans-activating (VP64) (panel B) domain driven from a PVD-specific promoter. (C) The transcriptionally repressive DAF-12 chimera cell-autonomously reduces dendrite growth in RD. ***P < 0.001, one-way ANOVA with Tukey post test. DBD, DNA-binding domain. (TIF) [file pbio.3000452.s003.tif]

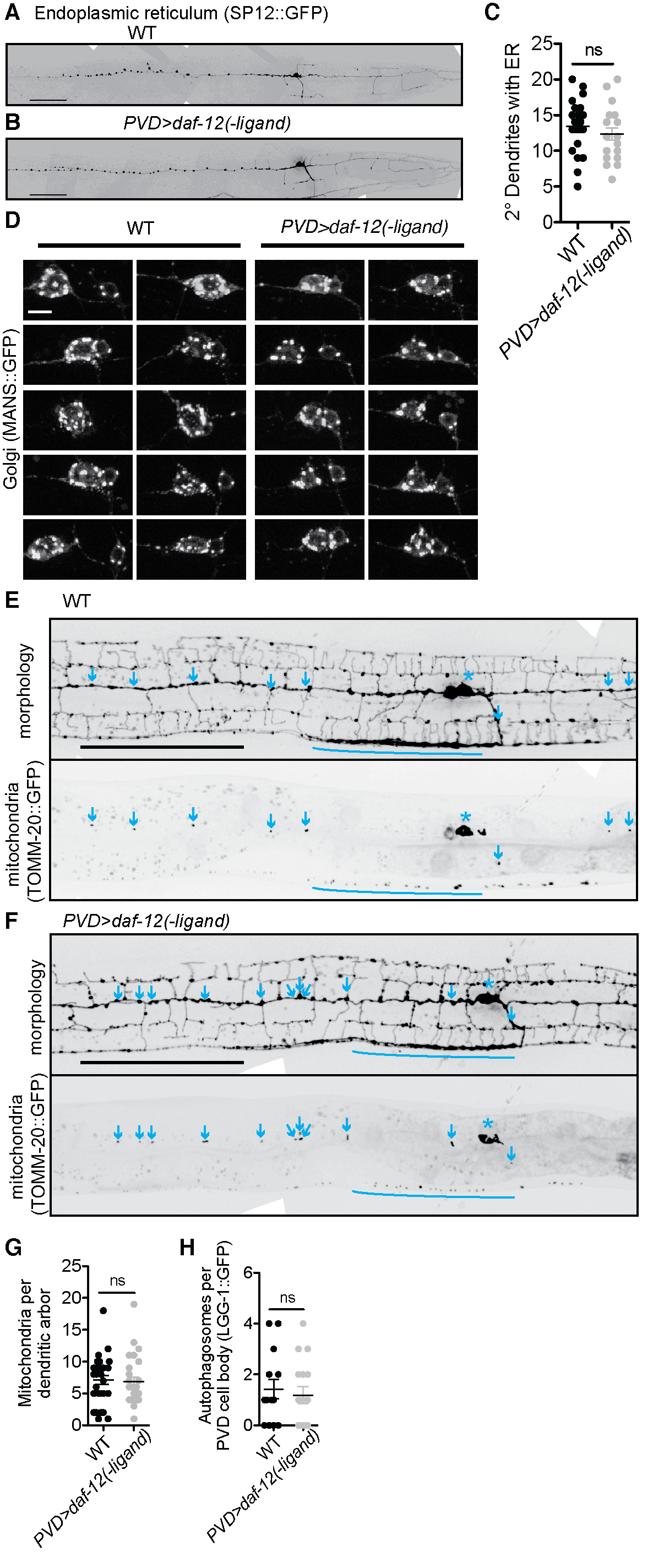

Supplement: S3 Fig — Endoplasmic reticulum morphology (A–C), Golgi morphology (D), mitochondria (E–G), and autophagy (H) all appear similar in wild type versus dauerized (PVD>daf-12(−ligand)) PVD. (A) Representative image of endoplasmic reticulum morphology in a wild-type day 1 adult: it fills the primary dendrite and a (seemingly arbitrary) subset of secondary and tertiary branches [1]. (D) Representative examples of the Golgi: it localizes exclusively, or nearly so, to the cell body and forms many small stacks. (E–F) Images of PVD morphology (PVD>myr-mCherry, top) and mitochondria (PVD>tomm-20::gfp, bottom), with blue arrows pointing to the locations of the dendritic mitochondria in each image. The cell body (blue asterisk) and axon (blue line) contain many mitochondria. In the PVD dendrite, there is no obvious correlation between mitochondria localization and dendritic branching. Scale = 50 um (A–B, E–F) or 5 um (D). (C, G–H) ***P < 0.001, two-tailed t test. 1. Liu X, Guo X, Niu L, Li X, Sun F, Hu J et al. Atlastin-1 regulates morphology and function of endoplasmic reticulum in dendrites. Nat Commun. 2019;10: 568. (TIF) [file pbio.3000452.s004.tif]

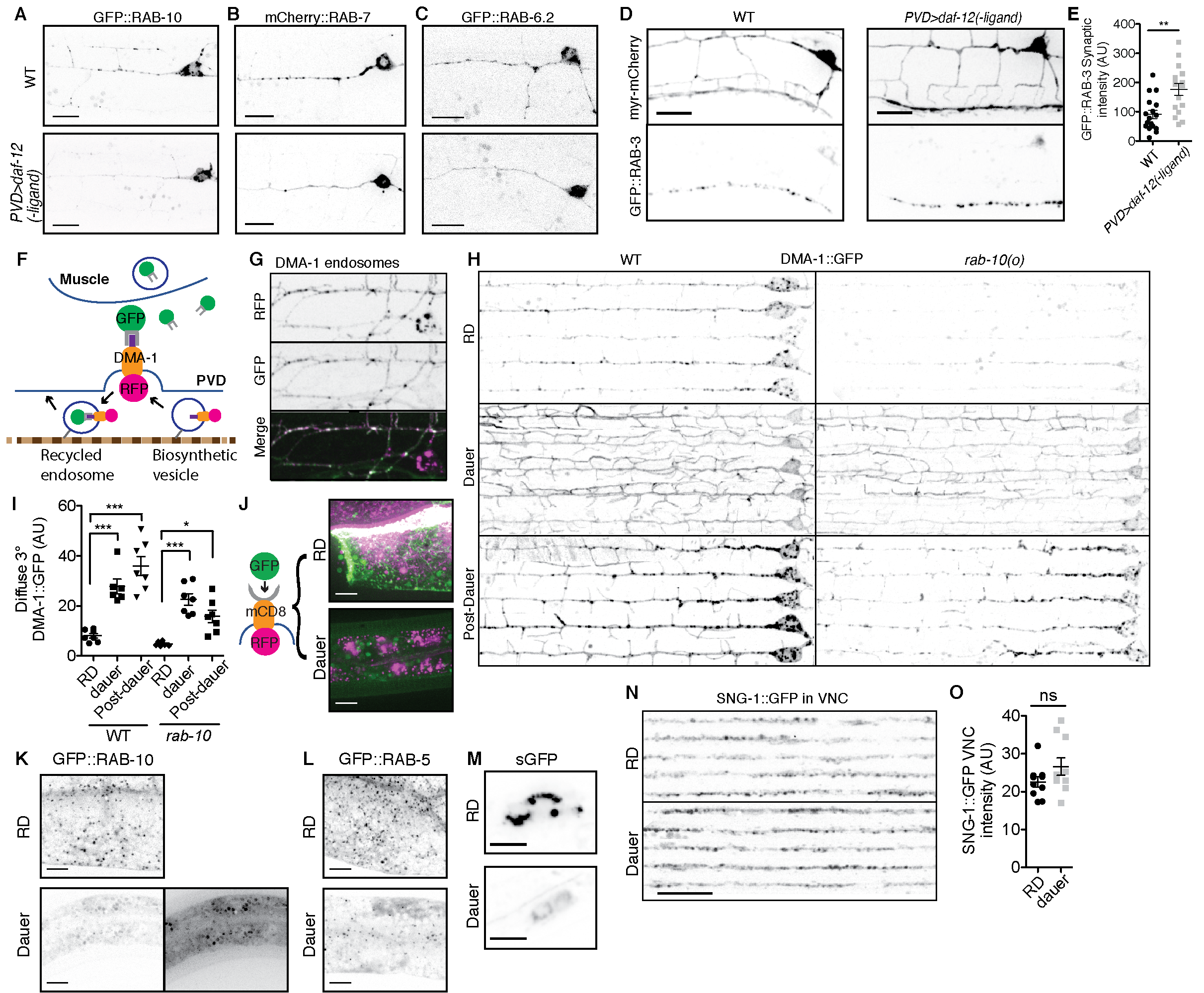

Supplement: S4 Fig — (A–C) Localization of RAB-10, RAB-7, and RAB-6.2 is less vesicular and more diffuse in dauerized dendrite compared to wild type. (D–E) Localization of RAB-3 is similar, but with increased accumulation, in dauerized versus wild-type PVD. **P < 0.01, two-tailed t test. (F) Strategy for constructing the DMA-1 guidance receptor endosome reporter. DMA-1 is fused to RFP on the cytosolic side and the GCN4 peptide epitope on the extracellular side. GFP fused to anti-GCN4 nanobody is secreted from the muscle. (G) Most DMA-1-containing vesicles in the PVD dendrite have been recycled off the plasma membrane. (H–I) DMA-1 exhibits both diffuse and endosomal localization in RD but is predominantly diffuse in dauer. In the rab-10 mutant, there is little endosomal DMA-1 in RD but much more diffuse DMA-1 in dauer, consistent with the model that RAB-10 is required to recycle endosomes to the plasma membrane and that endosome production is shut down in dauer. ***P < 0.001, one-way ANOVA with Tukey post test. (J–L) Example images of the intestinal and coelomocyte endocytosis reporters quantified in Fig 3O–3Q. The generic endosome reporter (J) expressed in the intestinal cells shows vesicular and tubular structures, many of which are co-labeled with endocytosed GFP. These tubular structures are characteristic of the recycling endosome compartment in these cells. In dauer, mCD8::RFP shows vesicular accumulations that are not co-labeled with GFP. (K) In RD, GFP::RAB-10 localizes to vesicles throughout the intestine, and these puncta are rarely observed in dauer, either when the imaged is processed in the same way as the RD image (left) or when the intensity is increased (right). (L) In RD, GFP::RAB-5 localizes to vesicles throughout the intestine, and vesicular localization is decreased in dauer. (M) Coelomocytes accumulate sGFP into endosomes in RD, and this accumulation is reduced in dauer. (N, O) Endogenously tagged Synaptogyrin/SNG-1::GFP shows punctate localization along the ventral [file pbio.3000452.s005.tif]

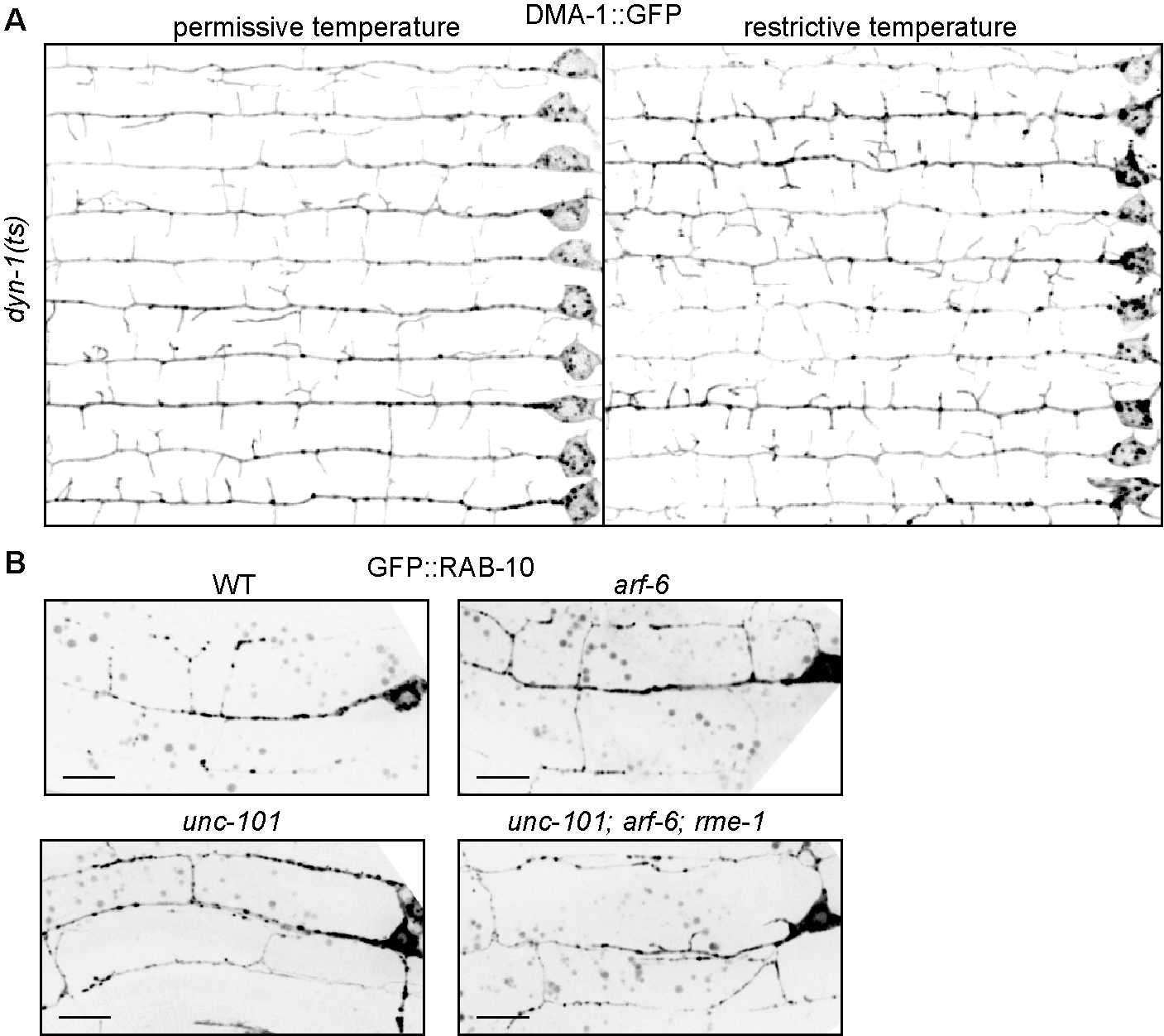

Supplement: S5 Fig — (A) Examples of DMA-1::GFP in the PVD anterior primary dendrite and cell body in the dynamin dyn-1(wy1150) mutant. This CRISPR-generated allele produces the same temperature-sensitive lesion as Drosophila shibire(ts1), at conserved reside G273D [1]. “Permissive temperature” worms were grown at 20°C, whereas “restrictive temperature” worms were shifted from 20°C to 30°C as early L4s for 5 hours prior to imaging. Note that there is no obvious reduction in the number of DMA-1::GFP-labeled vesicles along the primary dendrite at the restrictive temperature. There appears to be an increase in DMA-1::GFP intensity in the cell body at the restrictive temperature, consistent with the model in which Dynamin/DYN-1 is required to generate transport vesicles from the Golgi. (B) Loss-of-function alleles of known mediators of endocytosis and/or endosome recycling cause no obvious reduction in the number of GFP::RAB-10-labeled vesicles in the PVD dendrite. Endocytosis regulators examined are as follows: clathrin-independent endocytosis effector Arf6—arf-6(tm1447) (a deletion causing a putative null), clathrin adaptor AP-1μ—unc-101(wy50042) (G474A mutation relative to isoform a.2, causing W158Stop), and EpsI5-homology domain (EHD) protein—rme-1(b1045) (a deletion causing a putative null) [2]. 1. van der Bliek AM, Meyerowitz EM. Dynamin-like protein encoded by the drosophila shibire gene associated with vesicular traffic. Nature. 1991;351:411–414. 2. Grant B, Zhang Y, Paupard MC, Hall DH, Hirsh D, Lin SX. Evidence that RME-1, a conserved C. elegans EH-domain protein, functions in endocytic recycling. Nat Cell Biol. 2001;3: 573–579. (TIF) [file pbio.3000452.s006.tif]

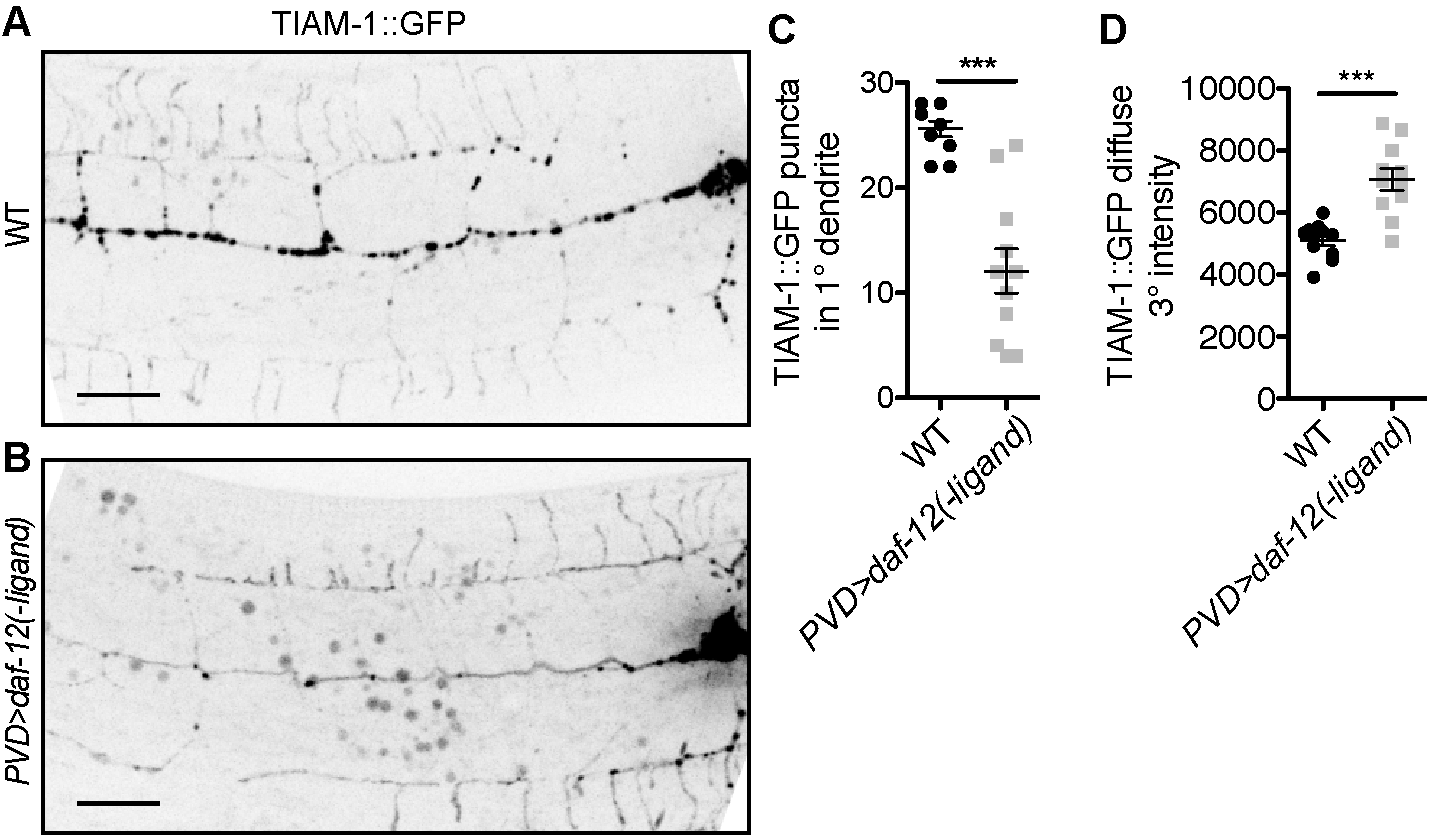

Supplement: S6 Fig — In wild-type RD, GFP::TIAM-1 localizes to vesicles throughout the dendrite (A, C), and it is less vesicular and more diffuse in dauerized (PVD>daf-12(−ligand)) PVD (B,D). Scale = 10 um. ***P < 0.001, two-tailed t test. (TIF) [file pbio.3000452.s007.tif]
